# A Logic Model for the Integration of Mental Health Into Chronic Disease Prevention and Health Promotion

**Published:** 2006-03-15

**Authors:** James Lando, Sheree Marshall Williams, Stephanie Sturgis, Branalyn Williams

**Affiliations:** National Center for Chronic Disease Prevention and Health Promotion, Centers for Disease Control and Prevention; National Center for Chronic Disease Prevention and Health Promotion, Centers for Disease Control and Prevention, Atlanta, Ga; National Center for Chronic Disease Prevention and Health Promotion, Centers for Disease Control and Prevention, Atlanta, Ga; National Center for Chronic Disease Prevention and Health Promotion, Centers for Disease Control and Prevention, Atlanta, Ga. Ms Williams is a Public Health Prevention Service Fellow

## Abstract

Mental illnesses such as depression or anxiety affect an individual's ability to undertake health-promoting behaviors. Chronic diseases can have a profound impact on an individual's mental health; in turn, mental health status affects an individual's ability to participate in treatment and recovery. A group of mental health and public health professionals convened to develop a logic model for addressing mental health as it relates to chronic disease prevention and health promotion. The model provides details on inputs, activities, and desired outcomes, and the designers of the model welcome input from other mental health and public health practitioners.

## Background

Many of the modifiable risk factors for chronic disease are behavioral, such as engaging in adequate physical activity, eating a healthy diet, and not using tobacco products. Mental illnesses such as depression or anxiety affect an individual's ability to undertake these health-promoting behaviors. Chronic diseases such as diabetes or cancer can have a profound impact on an individual's mental health; in turn, mental health status affects an individual's ability to participate in treatment and recovery. The cycle does not end with the patient. Family members and caregivers of people with chronic diseases are also affected psychologically, and they, too, may neglect their own health.

In January 2004, the National Center for Chronic Disease Prevention and Health Promotion convened "Mind the Body," a meeting of more than 50 Centers for Disease Control and Prevention (CDC) staff members and external experts. Most of the meeting participants were researchers, program managers, policy makers, and scientists who were involved in CDC surveillance, research, and programmatic activities addressing mental health and public health. The group discussed how to better integrate mental health into chronic disease prevention and health promotion efforts to accelerate an impact on public health. The group was particularly mindful of other federal agencies that have a mandate to address mental health research and programs, including the National Institute for Mental Health, the National Institute on Alcohol Abuse and Alcoholism, the National Institute on Drug Abuse, and the Substance Abuse and Mental Health Services Administration (SAMHSA).

The Mind the Body group concluded that a strong case could and should be made for explicitly addressing mental health as it relates to chronic disease prevention and health promotion. The group agreed that a detailed presentation of the types of inputs, activities, and desired outcomes of further work in this area would aid in building support, and the group decided to develop a logic model to accomplish this goal.

## Creating the Logic Model

Simply put, a logic model attempts to convey visually the connection between program activities and the program's desired outcomes; that is, the *logic* of the program. Many resources are available to public health practitioners who wish to develop logic models; some are referenced in the Resources section of the CDC Evaluation Working Group Web site, which is available from www.cdc.gov/eval.

In a series of meetings after Mind the Body, a small group explored ideas for a more deliberate and organized focus on the integration of mental health into public health. This group was assisted by CDC staff experienced in constructing logic models. A facilitator ensured that the group built an accurate and comprehensive model with an intelligible format. The facilitator also helped the group identify activities that would be undertaken, the intended outcomes, and the types of resources that would be needed. An initial model was circulated to selected CDC staff and a few external partners. The group then further refined and clarified its ideas — considering the addition of outcomes or activities and, more importantly, the order in which we expected outcomes to occur — until the draft of the logic model was ready for broader circulation. The Figure presents this draft.

**Figure:**
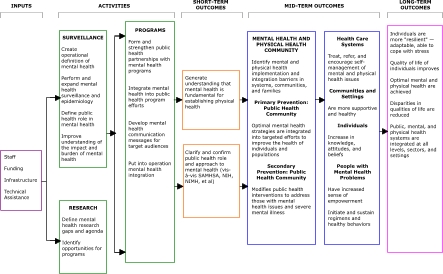
Draft of a logic model for integrating mental health into chronic disease prevention and health promotion.

This logic model does not necessarily represent official CDC policy. In addition, the model is a work in progress. As we circulate the model to people outside the Mind the Body group, we are likely to identify additional inputs, activities, or outcomes and will refine the model accordingly.

The intent of our logic model was to present the "big picture" in all its complexity. The model depicts the conceptual relationships between and among activities and outcomes, thus serving as a guide for discussions about what and how much to do. The logic model starts with inputs, or the resources needed to successfully undertake activities. If activities are successfully implemented, then a sequence of short-, mid-, and long-term outcomes will result.

### Inputs

The model's key inputs include dedicated staff, funding, and infrastructure in the form of an organizational home. Another resource, technical assistance, indicates that expertise outside of the areas of mental health and chronic disease prevention and health promotion would be required to provide guidance.

### Activities

Activities include surveillance, research, and programs. Surveillance is one of the underpinnings of public health. Creating operational definitions of mental health is an important step in mental health surveillance. These definitions need to acknowledge that mental health includes a broad spectrum of mental states ranging from optimal mental health to severe mental illness. Creating an operational construct for optimal mental health is not a trivial task, because although we may be able to agree on what constitutes mental illness based on conventional definitions, the question of whether people are performing at their peak mental health is quite another matter.

Strengthening surveillance for mental health status in multiple existing surveillance systems will improve our understanding of the interdependent relationships among mental health, risk factors, and physical health. It will also allow us to monitor trends across time and to make comparisons among different populations. It is important that surveillance be strategic and not duplicate activities others are performing.

CDC is already performing mental health surveillance and epidemiology. For example, the Behavioral Risk Factor Surveillance System (BRFSS) survey includes questions on quality of life and binge drinking. In 2006, BRFSS will field an optional module of 10 questions on symptoms of depression and lifetime diagnosis of depression and anxiety. To do so, CDC is collaborating with SAMHSA and its state mental health partners, and the BRFSS module promises to be a model of collaboration for future mental health surveillance activities.

Research on the relationship of mental health and chronic disease prevention and health promotion would benefit from a review of research gaps and the development of a research agenda to guide resource investment. Further research could build on intriguing findings from surveillance data and would ideally be geared toward identifying opportunities for programmatic activities to improve overall health.

For programmatic activities, partnerships should be forged that leverage the strengths of organizations and work on common goals. For example, CDC's Division of Adolescent and School Health convened an expert-panel meeting on mental health and health services to strengthen mental health offerings to departments of education. More work of this kind could be performed in any number of programmatic areas within chronic disease prevention and health promotion.

### Short-term outcomes

The logic model depicts our expectation that through enhanced surveillance, research, and programmatic activities in mental health, the public health community can generate understanding that good mental health is fundamental to maintaining good physical health. The model also indicates that the role of chronic disease prevention can be clarified and strengthened through partnerships.

### Mid-term outcomes

We identify several systems-level interventions in the health care delivery system, communities, and individuals that could lead to better mental health. In primary prevention, the public health community ensures that optimal mental health promotion strategies are integrated into other health promotion strategies to achieve maximum synergy. In secondary prevention, the public health community implements strategies to mitigate the impact of poor mental health on physical health risk behaviors that may lead to additional chronic disease comorbidities.

Additional mid-term outcomes include the following:

- Treatment, referral, and self-management of mental and physical health issues become the norm rather than the exception in clinical encounters.
- Workplaces, schools, and other communities become healthier and more supportive.
- Individuals have a better understanding of the integral link between mental and physical health.
- People with mental health problems gain a greater sense of empowerment to undertake and sustain healthy behaviors.

### Long-term outcomes

Ultimately, we hope that a concentrated effort to integrate mental and physical health activities will result in significant improvements in the health of the nation. We envision that individuals and communities will become more resilient — that is, better able to adapt to normal and extreme stressors. In targeting improved quality of life, we acknowledge that regardless of the life circumstances of individuals, quality of life should be independently targeted for intervention. In defining optimal physical and mental health as outcomes, we acknowledge that there is no health without mental health. And on a health care systems level, we expect that mental health, public health, and physical health care systems will be integrated at all levels to eliminate barriers to effective cooperation imposed by current systems.

## Conclusions

The logic model presented in this article is specific to the work of CDC in the area of chronic disease prevention and health promotion, but we hope that it is useful to others who would like to develop a more integrated mental and physical health focus in their public health programs.

This model depends on having a devoted locus of support, with sufficient funding and expertise. Without such support, we expect that the integration of mental health into chronic disease prevention will continue to progress slowly and incrementally. But if the activities we describe are undertaken, we would expect accelerated progress toward the goal of all people in an increasingly diverse society leading long, healthy, satisfying lives. By publishing this logic model, we hope to generate dialogue. We welcome your input as we move toward this future.

