# Supplementary material for: A Logic Model for the Integration of Mental Health Into Chronic Disease Prevention and Health Promotion
Source: Prev Chronic Dis. 2006 Mar 15;3(2):A61. (PMC1563949)

Figure. Draft of a logic model for integrating mental health into chronic disease prevention and health promotion.
SAMHSA indicates Substance Abuse and Mental Health Services Administration; NIH, National Institute for Health; NIMH, National Institute for Mental Health.

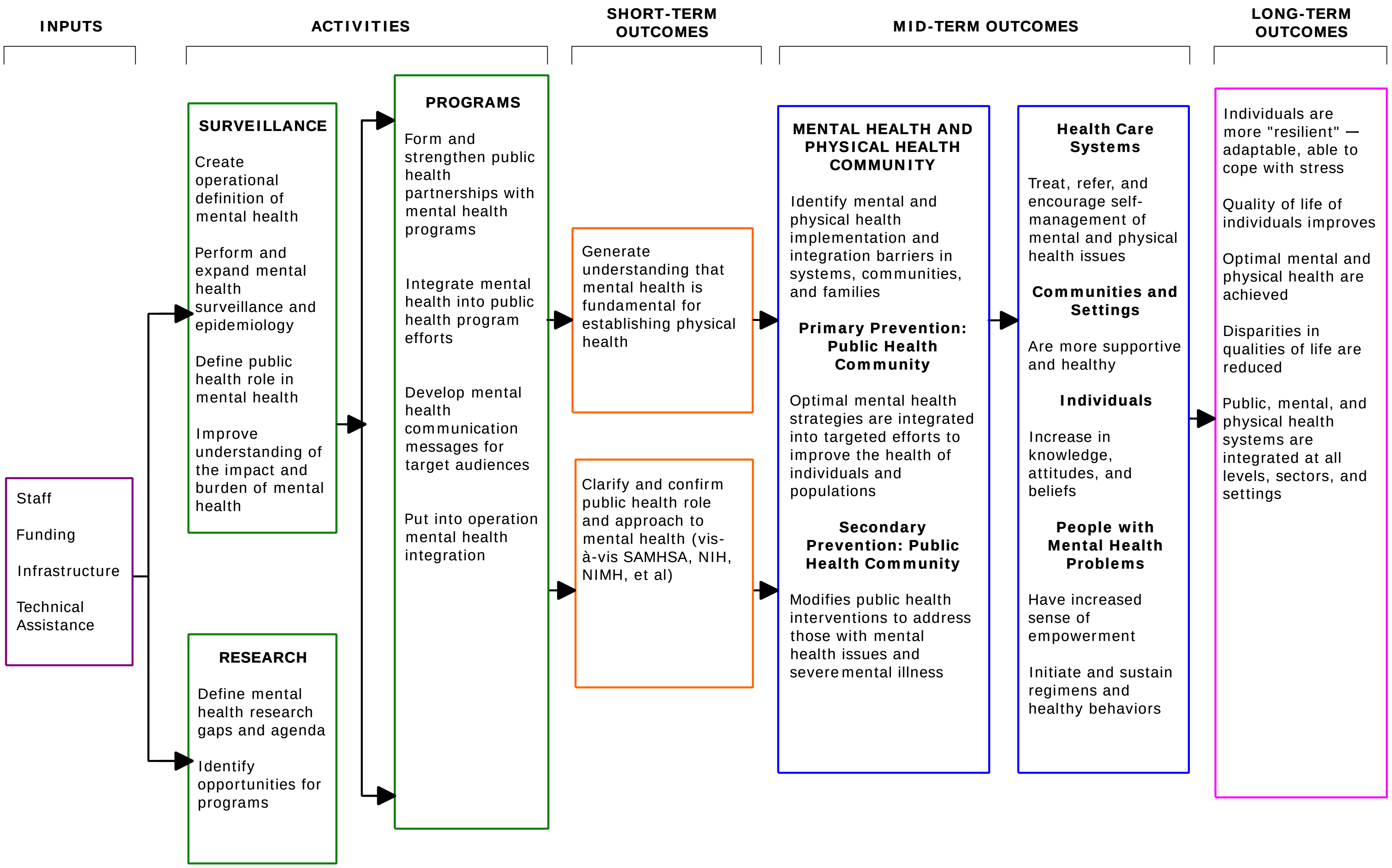

Supplement: Supplementary file 1 [file 05_0215_01.pdf]
